# Photobiomodulation therapy combined with carvedilol attenuates post-infarction heart failure by suppressing excessive inflammation and oxidative stress in rats

**DOI:** 10.1038/s41598-019-46021-1

**Published:** 2019-07-01

**Authors:** Vanessa Grandinetti, Fernando Pereira Carlos, Ednei Luiz Antonio, Helenita Antonia de Oliveira, Luis Felipe Neves dos Santos, Amanda Yoshizaki, Barbara Sampaio Dias Martins Mansano, Flávio André Silva, Leslie Andrews Porte, Gianna Móes Albuquerque-Pontes, Paulo de Tarso Camillo de Carvalho, Martha Trindade Manchini, Ernesto Cesar Leal-Junior, Paulo José Ferreira Tucci, Andrey Jorge Serra

**Affiliations:** 1 Universidade Nove de Julho, Programa de Pós-graduação em Biofotônica Aplicada as Ciências da Saúde, São Paulo, Brazil; 2 Universidade Federal de São Paulo, Programa de Pós-graduação em Cardiologia, São Paulo, Brazil; 3 Universidade Adventista de São Paulo, São Paulo, Brazil

**Keywords:** Regenerative medicine, Heart failure, Cardiac device therapy

## Abstract

The post-myocardial infarction heart failure (HF) still carries a huge burden since current therapy is unsuccessful to abrogate poor prognosis. Thus, new approaches are needed, and photobiomodulation therapy (PBMt) may be a way. However, it is not known whether PBMt added to a standard HF therapy provides additional improvement in cardiac remodeling in infarcted rats. This study sought to determine the combined carvedilol-drug and PBMt with low-level laser therapy value in HF. Rats with large infarcts were treated for 30 days. The functional fitness was evaluated using a motorized treadmill. Echocardiography and hemodynamic measurements were used for functional evaluations of left ventricular (LV). ELISA, Western blot and biochemical assays were used to evaluate inflammation and oxidative stress in the myocardium. Carvedilol and PBMt had a similar action in normalizing pulmonary congestion and LV end-diastolic pressure, attenuating LV dilation, and improving LV systolic function. Moreover, the application of PBMt to carvedilol-treated rats inhibited myocardial hypertrophy and improved +dP/dt of LV. PBMt alone prevented inflammation with a superior effect than carvedilol. Carvedilol and PBMt normalized 4-hydroxynonenal (a lipoperoxidation marker) levels in the myocardium. However, importantly, the addition of PBMt to carvedilol attenuated oxidized protein content and triggered a high activity of the anti-oxidant catalase enzyme. In conclusion, these data show that the use of PBMt plus carvedilol therapy results in a significant additional improvement in HF in a rat model of myocardial infarction. These beneficial effects were observed to be due, at least in part, to decreased myocardial inflammation and oxidative stress.

## Introduction

Myocardial infarction is one of the common causes of heart failure (HF)^1^. Myocardial reperfusion is the most effective therapy to reduce the deleterious effects of myocardial infarction and preserver cardiac performance into the acute setting^2^. Although, the procedure of myocardial reperfusion has been optimized by advances in primary percutaneous coronary intervention and new drugs delay the progression of the disease, a significant number of patients develop HF with considerable morbidity and mortality^2,3^. Thus, there is a need for new cardioprotective approaches to mitigate post-infarction HF.

We and other researcher groups have demonstrated a smaller myocardial injury and attenuated left ventricular (LV) dysfunction in rats submitted to photobiomodulation therapy (PBMt)^4–6^. Although these data are stimulating, there are unclear issues that must be resolved prior to a clinical trial. In this regard, studies have only assessed the PBMt role at the early infarction stage; thereby, there is a lack of knowledge on the potential usefulness of PBMt in the course of HF^7^. Furthermore, there is no information on whether PBMt added to a standard HF therapy provides further improvement in cardiac remodeling in infarcted rats.

Therefore, we evaluated the combined effect of carvedilol and PBMt with low-level laser therapy in attenuating post-ischemic HF. The choice of carvedilol was based on clinical and experimental studies demonstrating improved post-infarction cardiac remodeling^8,9^. Moreover, carvedilol and PBMt have an analogous effect on inflammation and oxidative stress^7–11^, in which it could lead to a hypothesis of the synergistic effect.

## Results

**Figure 1:**
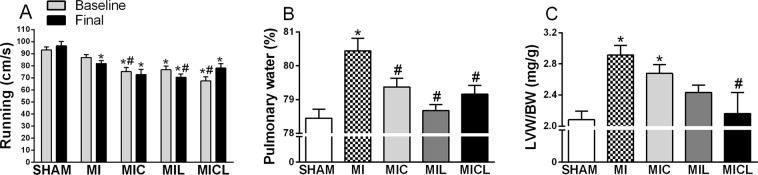
Effects of long-term treatment with carvedilol, PBMt, and combined therapy on functional fitness (**A**), pulmonary congestion (**B**), and LV mass (**C**) in infarcted rats compared with those in sham-operated rats. *p < 0.05 versus the sham group for the respective time. LVW/BW, LV mass/body weight. ^#^p < 0.05 versus the MI group for the respective time.

Functional fitness was significantly decreased in all infarcted rats with no therapeutic effect (Fig. 1A). However, pulmonary congestion was reduced with therapies (Fig. 1B), and the MICL group had a normalized LV mass/body weight ratio (Fig. 1C).

**Figure 2:**
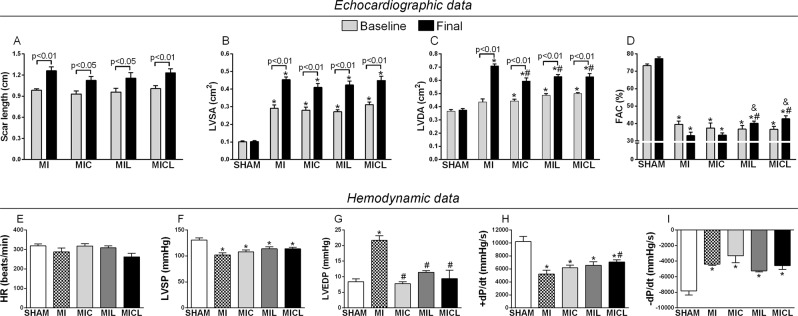
Echocardiographic and hemodynamic analysis of LV in infarcted rats treated with carvedilol, PBMt, and combined therapy. (**A**) Length of infarct scar; (**B**) LVDA, diastolic area; (**C**) LVSA, systolic area; (**D**) FAC, fractional area change. *p < 0.01 versus the sham group for the respective time. ^#^p < 0.05 versus the MI group for the respective time.

Infarction size and systolic area were similar in both MI groups (Fig. 2A,B) on echocardiography, but a minor diastolic area and improved systolic performance were found in the MIL and MICL groups (Fig. 2C,D). On LV hemodynamic examination, heart rate was similar between groups, but there was a reduction in LV pressure in all infarcted rats (Fig. 2E,F). Infarcted rats had overt LV dysfunction as indicated by an increased LV end-diastolic pressure (LVEDP) and decreased +dP/dt and -dP/dt (Fig. 2G–I). However, carvedilol and PBMt has normalized the LVEDP, and combined therapy had additive effect to improve +dP/dt.

**Figure 3:**
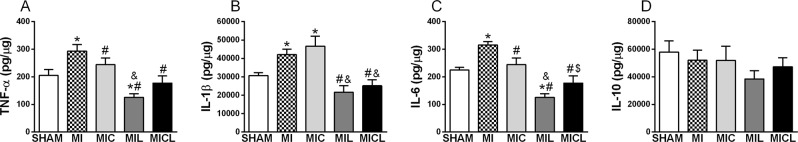
Effects of carvedilol, PBMt, and combined therapy in myocardial inflammation. (**A**) TNF-α, tumor necrosis factor alpha; (**B**) IL-1β, interleukin 1 beta; (**C**) IL-6, interleukin 6; (**D**) IL-10, interleukin 10. *p < 0.05 versus the sham group. ^#^p < 0.05 versus the MI group. ^&^p < 0.05 versus the MIC group.

The TNF-α, IL-1β, and IL-6 cytokines levels were elevated in the MI group (Fig. 3A–C), in which carvedilol alone has reduced TNF-α and IL-6 levels. Notwithstanding, PBMt alone or combined with carvedilol has normalized cytokine content. There was no change in the expression of the anti-inflammatory IL-10 cytokine (Fig. 3D).

**Figure 4:**
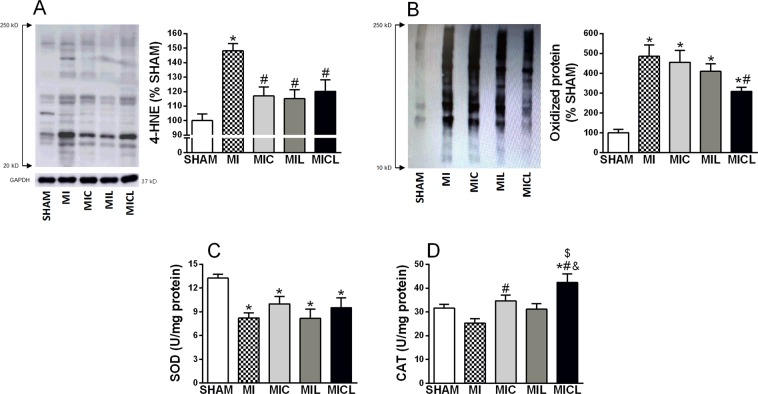
Effects of carvedilol, PBMt, and combined therapy on myocardial oxidative stress. (**A**) 4-HNE, 4-hydroxynonenal; (**B**), oxidized protein; (**C**) SOD, superoxide dismutase; (**D**) CAT, catalase. *p < 0.05 versus the sham group. ^#^p < 0.05 versus the MI group. ^&^p < 0.05 versus the MIC group.

The 4-hydroxynonenal was used as a lipoperoxidation marker and was significantly higher in the MI group (Fig. 4A). This increase in lipoperoxidation was prevented by all treatments. Infarcted rats showed a significant increase in oxidized protein content, in which there was attenuation only in the MICL group (Fig. 4B). SOD activity was significantly decreased in infarcted rats, and there were no beneficial therapy effects (Fig. 4C). Catalase activity had not been altered in the MI group but had shown some increase in the MIC group. In fact, an increased catalase activity was found only in the combination therapy (Fig. 4D).

## Discussion

This is the first study to show that the combination of carvedilol-drug therapy and PBMt may relieve post-infarction HF. There was a noticeable benefit of combined therapy in attenuating myocardial hypertrophy, LV dysfunction, and pulmonary congestion. These findings were associated with a significant reduction in myocardial inflammation and oxidative stress.

Reduced physical fitness is a well-established disorder in HF^12^, and previous reports have shown the doubtful effect of beta-blockers. Thereby, there are data illustrating physical fitness improvement and the null effect of carvedilol^13,14^. In contrast, studies evaluating the role of PBMt in physical fitness are rare. To our knowledge, a single study that applied light-emitting diode in the gastrocnemius muscle of infarcted rats has reported improvement in peak treadmill speed^15^. Hence, although we have found amelioration in cardiac remodeling, this would not be enough to increase physical performance, in which intrinsic skeletal muscle abnormalities seem to play a prominent role in exercise intolerance^16^.

Our data indicated that, in infarcted rats, carvedilol has been effective in congestive HF, causing beneficial changes in pulmonary water content, LV dilation, and LVEDP. The benefits of PBMt similar to those of carvedilol, including normalization of myocardial mass and improved LV fractional area change. In fact, a growing body of evidence supporting the use of PBMt to improve post-infarction HF over a range of animal species (e.g. rats and dogs)^7,17,18^. Moreover, evidence of accelerated wound healing in cardiomyocyte tissue after low-level laser therapy in patients undergoing myocardial revascularization surgery seems to suggest PBMt as a promising clinical approach^19^. The similar effect of carvedilol and laser on cardiac remodeling led to hypothesize that increased cardioprotection could be achieved by combining the therapies. Thus, the rats exhibited a more pronounced effect on myocardial mass and systolic LV performance.

To assess putative mechanisms linked to cardioprotection, we focused on inflammation and oxidative stress, which are both assumed as important pathophysiological events in HF^20,21^. In this regard, we have confirmed that carvedilol reduces post-infarction inflammation^10^, as illustrated by TNF-α and IL-6 expression. Nevertheless, PBMt has shown a more pronounced anti-inflammatory effect by reducing all pro-inflammatory cytokines (i.e. TNF-α, IL-1β, and IL-6). These findings extend that of a previous study of our group, in which the PBMt was shown to have an anti-inflammatory action in the early infarction stage^4^. The reduction in inflammation is an important mechanism to attenuate the remodeling of the extracellular matrix as well as pro-fibrogenic stimulus post-infarction^10,22^.

Carvedilol therapy and PBMt protected the myocardium from oxidative stress, as assessed by the level of 4-hydroxynonenal, one of the major end products of lipoperoxidation. Homeostasis of 4-hydroxynonenal has a key role in cardiac remodeling because increased aldehydic load leads to impaired mitochondrial metabolism^23^. This has been highlighted in studies that found an association between improved LV function and reduced cardiac 4-hydroxynonenal adducts in rats and patients with heart disease^23,24^. Our findings demonstrated that the myocardial antioxidant effects appear to be mediated by catalase (see Fig. 3), in which it decomposes hydrogen peroxide before it can cause cellular damage^25^. Furthermore, catalase activity was higher with the combination of carvedilol and PBMt, which could partially explain our finding of reduced oxidized protein only in the MICL group.

In conclusion, this experimental study shows that the PBMt adds benefits to carvedilol in attenuating post-infarction HF in terms of attenuated myocardial hypertrophy, increased LV performance, and reduced inflammation as well as oxidative stress.

### Limitations

A limitation of the present study is whether PBMt could also induce benefits when combined with other beta-blockers or drugs standardized in HF (e.g., angiotensin-converting enzyme inhibitors). In addition, we have treated the animals with a target dose of 19.998 J, and therefore, it is likely that the best irradiation dose should be further clarified.

## Methods

### Experimental groups

The research was approved by the Institutional Research Ethics Board of Nove de Julho University (process: 0016/2016), and all methods were performed in accordance with the relevant guidelines and regulations. Experiments were performed under anesthesia with ketamine (50 mg/kg)/xylazine (10 mg/kg) mixture. Female Wistar rats (200–250 g; aged 12 weeks) were enrolled to infarcted rats non-treated (MI) or submitted to carvedilol (MIC), PBMt (MIL), and combination therapy (MICL).

### Myocardial infarction model

The surgical procedure to induce chronic infection was performed according to a well-established technique^4,5,26^. Under anesthesia and artificial ventilation 155 with a Harvard Rodent Ventilator (Model 863; Harvard Apparatus, Holliston, MA), a left thoracotomy was performed to externalize the heart, and the coronary artery ligated with 6-0 polypropylene. The heart was quickly returned to its position and the thorax immediately closed. Sham rats were submitted to a similar procedure, with the exception of coronary occlusion.

### Treatments

Carvedilol was provided by Baldacci (São Paulo, Brazil) and administered with water as on a well-reported dose (10 mg/kg/day) to attenuate cardiac remodeling^10,27^. The DMC Thera Laser aluminum indium gallium phosphorus – AlGaInP (DMC, São Carlos, SP, Brazil) was used for the irradiation under the parameters listed in Table 1.

**Table 1:** PBMt parameters.

Points	3
Irradiation per point	1
Wavelength (nm)	830
Output power (mW)	100
Laser beam (cm^2^)	0.028
Time per point (seg)	66.66
Total energy (J)	19.998
Fluence (J/cm^2^)	714
Irradiance (W/cm^2^)	3.57

**Figure 5:**
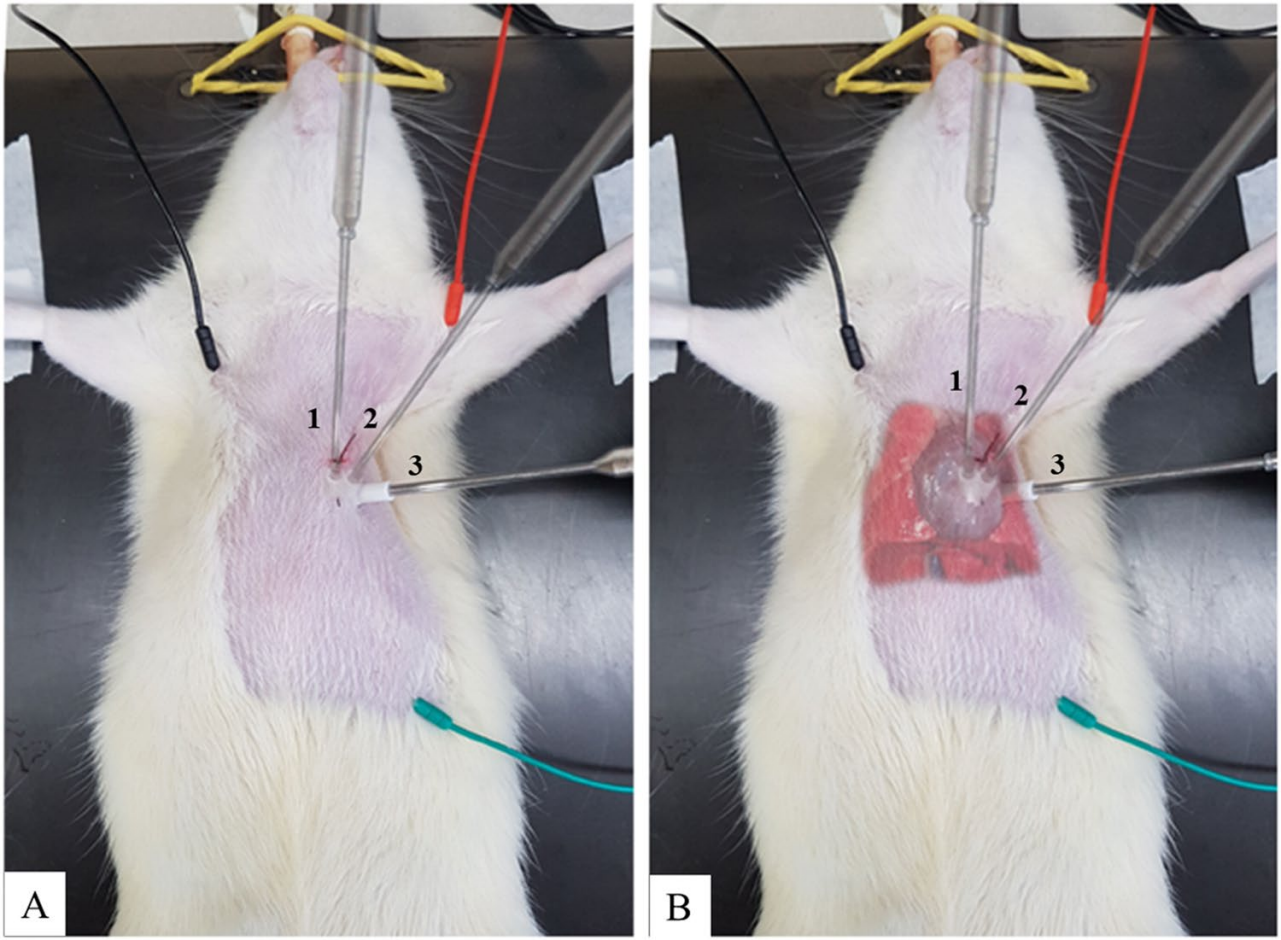
PBMt application at three thoracic points (Panel A). In Panel B, it is possible to visualize the position of the heart within the thorax in relation to the positioning of the bean laser.

The laser was transthoracically applied three days a week, while the irradiation was performed at three anatomical locations for a duration of 66.66 per point. Laser beam was placed in contact with the thorax surface corresponding to the points that made it possible to reach the heart (Fig. 5). The Sham, MI, and MIC groups were submitted to a similar PBMt procedure, yet the device was not used. Carvedilol and PBMt were started after the coronary occlusion and continued for 30 days.

### Functional fitness

Functional fitness was evaluated on the 7th and 29th day of the study by using a motorized treadmill^26^. Each rat underwent a 2-minute warm-up period at 25 cm/s, following which the running speed was increased by 9 cm/s every 2 min till the rats were exhausted.

### LV performance

As previously described^5^, transthoracic echocardiographic was performed using an HP Sonos-5500 (Hewlett Packard, Andover, MA, USA) echocardiography. Rats were imaged following 2- and 30-days post-infarction, in which only rats with large infarcts ( ≥ 37% of LV) were included. Immediately after the last echocardiography, the rats were intubated, ventilated (rodent ventilator, model 683, Harvard Apparatus, Holliston, MA, USA) and a 2-F gauge Millar catheter-tip micromanometer SPR-320 (Millar Instruments, Houston, TX, USA) was inserted through the right carotid artery into the LV cavity^4^. Measurements of LV parameters, including heart rate (HR), LV systolic pressure (LVSP), LV end-diastolic pressure (LVEDP), and maximal positive ( + dP/dt) and negative (-dP/dt) time derivatives of the developed pressure were studied using AcqKnowledge 3.5.7 software (Biopac Systems Inc., Santa Barbara, CA, USA).

### Enzyme-linked immunosorbent assay (ELISA)

Frozen remote myocardium was homogenized in phosphate-buffered saline plus proteinase inhibitor cocktail (Sigma Chemical, St Louis, MO, USA). Homogenates were subjected ELISA using the specific commercial kit (R&D Systems, USA) to evaluate Tumor necrosis factor alpha (TNF-α), Interleukin 1 beta (IL-1β), Interleukin 6 (IL-6), and Interleukin 10 (IL-10).

### Western blot

The frozen remote myocardium was homogenized as previously described^28^, and 20 μg of the homogenates were prepared for transfer onto hydrophobic polyvinylidene membranes (Hybond-P, Amersham Biosciences; Piscataway, NJ, USA)^28^. The membranes were incubated overnight at 4 °C with rabbit anti-4-HNE (1:2000 dilution; Abcam, Cambridge, MA, USA). Then, membranes were washed five times and incubated for 60 min with horseradish peroxidase-conjugated goat anti-rabbit secondary antibody (1:2000; Invitrogen, San Diego, CA, USA). Bound antibody was detected by using chemiluminescence, and bands were imaged by using Amersham Imager 600 system (GE Health Care, Little Chalfont, UK, USA).

### Protein oxidation

Carbonyl groups inserted into proteins by oxidative reactions were evaluated with Abcam kit ab178020 (Abcam, Cambridge, MA, USA) for an equal protein load (20 μg)^24^. The samples were then loaded onto SDS PAGE gels and DNP conjugated proteins were detected by western blotting using primary DNP antibody and HRP conjugated secondary antibody. Bound antibody was detected by using chemiluminescence, and bands were imaged by using Amersham Imager 600 system (GE Health Care, Little Chalfont, UK, USA).

### Antioxidant enzymes

Muscles (~ 50 mg) were homogenized in phosphate buffer (0.1 M; pH 7.4), and samples were centrifuged twice for 15 min each (800 and 13400, xg). Catalase (CAT) activity was assessed by mixing the homogenates with 10 mM H_2_O_2_ (10% v/v) and 50 mM phosphate buffer (90% v/v); thereby, the decrease of H_2_O_2_ over 5 min at 30 °C was measured in 240 nm. Superoxide dismutase (SOD) activity was evaluated by adding nitro blue tetrazolium, βNADH and phenylmethosulfate, and measuring the absorbance for 5 min at 30 °C at 560 nm.

### Statistical analysis

Statistical analyses were performed with GraphPad Prism 5.0 (CA, USA). ANOVA (Bonferroni post hoc test) was applied to evaluate Gaussian data, and Kruskal-Wallis (Dunn’s post hoc) test was applied to analyze nonnormality data. Results were expressed as mean ± SEM, and a P-value ≤ 0.05 was considered statistically significant.

## Data Availability

The datasets generated and analyzed during the current study are available from the corresponding author on reasonable request.
